# Prenatal Diagnosis of Alobar Holoprosencephaly With Cyclopia and Proboscis: A Case Report

**DOI:** 10.7759/cureus.116592

**Published:** 2026-09-20

**Authors:** Manisha Mahajan, Varun Mahajan, Chandla Nikita Kishor Singh

**Affiliations:** 1 Department of Radiology, Government Medical College, Udhampur, IND; 2 Department of General Surgery, Government Medical College, Jammu, IND; 3 Department of Anesthesia and Critical Care, Government Medical College, Jammu, IND

**Keywords:** alobar holoprosencephaly, congenital anomaly, cyclopia, prenatal diagnosis, prenatal ultrasound, proboscis

## Abstract

Alobar holoprosencephaly (HPE) is a severe developmental abnormality of the fetal brain. It can also be associated with major facial abnormalities. We report a case diagnosed during a routine ultrasound scan at 22 weeks of pregnancy in a 26-year-old G2P1L1 woman. Ultrasound showed a single ventricular cavity, an absent interhemispheric fissure and falx cerebri, and fused thalami. Facial examination showed a single median orbit and a midline proboscis, consistent with cyclopia. No other structural abnormalities were seen. After counseling about the severe nature and poor prognosis of the condition, the pregnancy was terminated at 23 weeks. This case highlights the importance of examining the fetal face when alobar HPE is suspected on ultrasound.

## Introduction

Holoprosencephaly (HPE) is a developmental abnormality of the fetal brain. It occurs when the forebrain does not divide normally during early development. HPE is classified into three forms: alobar, semilobar, and lobar. Alobar HPE is the most severe form. It is associated with failure of separation of the cerebral hemispheres, a single ventricular cavity, absence of midline brain structures, and fusion of the thalami [1]. Facial abnormalities can occur with HPE. Their severity can vary. They include cleft lip and palate, proboscis, and cyclopia. Cyclopia is one of the most severe facial abnormalities. It is characterized by a single median orbital structure [2]. Prenatal ultrasound is important for detecting HPE. The main findings of alobar HPE include a monoventricle, absence of the interhemispheric fissure and falx cerebri, and fused thalami [3]. Examination of the fetal face can provide additional information. Cyclopia and proboscis can also be detected on prenatal ultrasound [4]. Three-dimensional ultrasound may be useful when facial abnormalities are complex and need further assessment [5]. Early diagnosis is important because severe HPE has a poor prognosis. It also allows proper counseling and discussion of pregnancy-management options [6]. We report a case of alobar HPE with cyclopia and proboscis diagnosed during a routine anomaly scan at 22 weeks of gestation.

## Case presentation

A 26-year-old G2P1L1 woman underwent a routine second-trimester anomaly scan at 22 weeks of gestation. There were no significant problems during the pregnancy before the ultrasound examination. There was no history of diabetes mellitus, hypertension, thyroid disease, epilepsy, or other significant maternal medical illness. There was no history of exposure to known teratogenic medications, alcohol, tobacco, or other potentially harmful substances during pregnancy. There was no history of consanguineous marriage or family history of congenital anomalies, HPE, or other known genetic disorders. Her previous pregnancy resulted in a live birth without any reported congenital anomaly or major pregnancy-related complication. A first-trimester ultrasound was done, which was reported as unremarkable, with no major structural anomaly identified. No aneuploidy screening was performed. Her routine antenatal blood tests were apparently normal, and no significant abnormality was seen until the second-trimester anomaly scan. Ultrasound revealed marked abnormalities of the cerebral midline. The interhemispheric fissure and falx cerebri were not visualized. It had a single huge ventricle, or monoventricle. The thalami were fused. Failure of normal separation of cerebral hemispheres was also observed. These findings were compatible with alobar HPE (Figures 1A, 1B). The fetal face was then examined carefully. One median orbital structure was seen. This was in agreement with cyclopia (Figure 1C). The nose was absent, and a midline proboscis was present (Figures 1D, 1E). The findings were consistent with the diagnosis of alobar HPE with cyclopia and proboscis (Figures 1C-1E). A detailed fetal anomaly scan was done. Cardiac, thoracic, abdominal, renal, spinal, and skeletal structures were evaluated and reported as normal. No other structural abnormalities were seen. Counseling was given to the parents regarding the brain and facial abnormalities and the poor prognosis. The possibility of an underlying chromosomal or genetic abnormality was also raised. Genetic testing was offered and discussed with the parents but was not performed. Options for management of pregnancy were discussed, and after counseling, they opted for termination of pregnancy at 23 weeks gestation. The ultrasound findings were characteristic enough to make the diagnosis, and fetal MRI was not performed as it was not considered necessary for additional diagnostic information. Following termination of pregnancy at 23 weeks' gestation, no fetal examination or postmortem assessment was carried out. Therefore, the prenatal ultrasound diagnosis was not pathologically confirmed.

**Figure 1:**
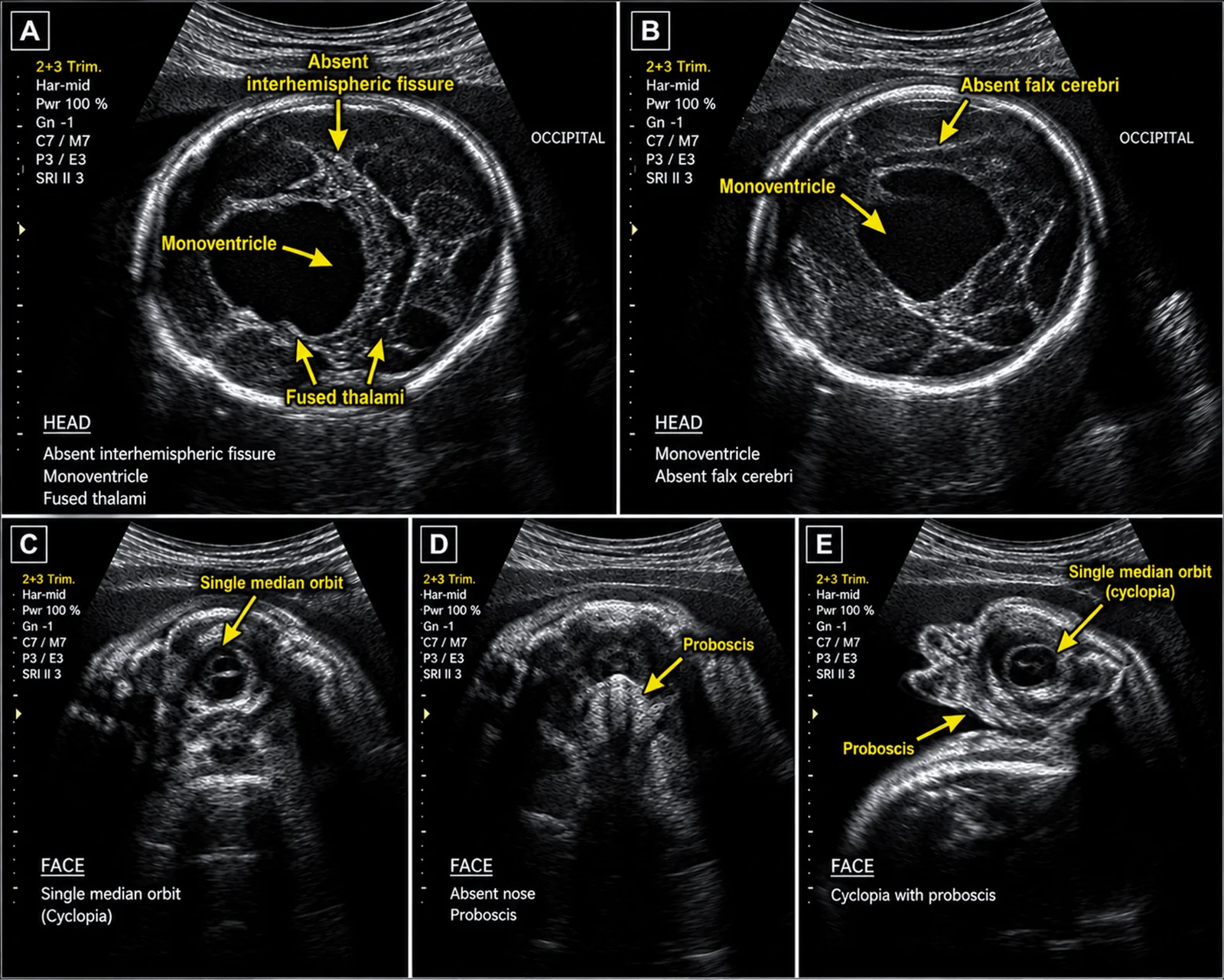
Prenatal ultrasonographic features of alobar holoprosencephaly with cyclopia and proboscis. (A) Cranial ultrasound showing absence of the interhemispheric fissure, a monoventricle, and fused thalami. (B) Cranial ultrasound showing a monoventricle and absence of the falx cerebri. (C) Facial ultrasound showing a single median orbital structure consistent with cyclopia. (D) Facial ultrasound showing an absent nose with a midline proboscis (arrow). (E) Facial ultrasound showing cyclopia with proboscis

## Discussion

The most severe type of HPE is alobar HPE. It occurs as a result of improper development and separation of the forebrain. Normally, the embryonic forebrain undergoes midline cleavage and separates into two cerebral hemispheres. Failure of this normal separation results in varying degrees of cerebral malformation, with alobar HPE representing the most severe form. The associated disturbance of midline development can also affect facial formation, resulting in severe abnormalities such as cyclopia and proboscis. The disorder typically leads to poor outcomes. The typical sonographic characteristics of alobar HPE were described by Filly et al., who also showed the prenatal accuracy of the diagnosis [6]. As a result, ultrasonography is very helpful for early diagnosis, especially during a routine anomaly scan. Fetal MRI may be helpful when ultrasound findings are unclear or when more detail about the fetal brain is needed [7]. In our case, MRI was not necessary because the ultrasound findings were sufficiently characteristic for the diagnosis and were not expected to provide additional diagnostic information. Chromosomal abnormalities and mutations in genes such as SHH, ZIC2, SIX3, and TGIF1 have been described, suggesting that alobar HPE may have a genetic basis [8]. Therefore, genetic counseling should be provided following diagnosis. In addition to helping determine the underlying cause, genetic testing may help with counseling regarding future pregnancies. We discussed and offered genetic testing to the parents in our case, but it was not carried out. Severe facial deformities are also associated with alobar HPE [9]. One of the most serious facial deformities is cyclopia, which may be associated with a midline proboscis. These facial deformities are typically associated with a poor prognosis and indicate the severity of the underlying disruption of midline development [10]. In this case, the severe craniofacial phenotype of HPE was clearly demonstrated by the combination of the typical cerebral features of alobar HPE with cyclopia and a midline proboscis. The clinical significance of this case extends beyond the well-established capability of ultrasound to diagnose alobar HPE. Instead, it emphasizes how crucial it is to carefully examine the fetal face following the discovery of a significant midline brain abnormality. Diagnosing cyclopia and proboscis is important for parental counseling and provides further information on the severity of the condition. In our case, the parents decided to terminate the pregnancy at 23 weeks of gestation after receiving counseling regarding the poor prognosis and available pregnancy-management options following the identification of the combined brain and facial abnormalities. In our case, we did not perform postmortem evaluation or genetic testing. As a result, there was no pathological or genetic confirmation. Although the prenatal ultrasound findings were typical of alobar HPE with cyclopia and proboscis, the absence of genetic and pathological confirmation is an important limitation of this report.

## Conclusions

During prenatal ultrasound, typical brain findings can help diagnose alobar HPE. Facial abnormalities such as cyclopia and proboscis can provide additional information about the severity of the condition. Carefully examining both the fetal brain and face is important when a midline brain abnormality is suspected. Early diagnosis helps counsel parents and make appropriate pregnancy-management decisions.

## References

[1] Dubourg C, Bendavid C, Pasquier L, Henry C, Odent S, David V (2007). Holoprosencephaly. Orphanet J Rare Dis.

[2] Kauvar EF, Muenke M (2010). Holoprosencephaly: recommendations for diagnosis and management. Curr Opin Pediatr.

[3] Greene MF, Benacerraf BR, Frigoletto FD Jr (1987). Reliable criteria for the prenatal sonographic diagnosis of alobar holoprosencephaly. Am J Obstet Gynecol.

[4] Schinzel A, Savoldelli G, Briner J, Schmid W (1984). Prenatal ultrasonographic diagnosis of holoprosencephaly. Two cases of cebocephaly and two of cyclopia. Arch Gynecol.

[5] Lai TH, Chang CH, Yu CH, Kuo PL, Chang FM (2000). Prenatal diagnosis of alobar holoprosencephaly by two-dimensional and three-dimensional ultrasound. Prenat Diagn.

[6] Filly RA, Chinn DH, Callen PW (1984). Alobar holoprosencephaly: ultrasonographic prenatal diagnosis. Radiology.

[7] Kousa YA, du Plessis AJ, Vezina G (2018). Prenatal diagnosis of holoprosencephaly. Am J Med Genet C Semin Med Genet.

[8] Dubourg C, Lazaro L, Pasquier L (2004). Molecular screening of SHH, ZIC2, SIX3, and TGIF genes in patients with features of holoprosencephaly spectrum: mutation review and genotype-phenotype correlations. Hum Mutat.

[9] Raman R, Mukunda Jagadesh G (2014). Antenatal diagnosis of alobar holoprosencephaly. Case Rep Radiol.

[10] Raam MS, Solomon BD, Muenke M (2011). Holoprosencephaly: a guide to diagnosis and clinical management. Indian Pediatr.

